# Moral orienting systems: reconceptualizing moral injury as moral disorientation

**DOI:** 10.3389/fpsyt.2026.1733645

**Published:** 2026-02-27

**Authors:** Zachary Moon, L. Callid Keefe-Perry

**Affiliations:** 1 Brite Divinity School, Fort Worth, TX, United States; 2 Clough School of Theology and Ministry, Boston College, Chestnut Hill, MA, United States

**Keywords:** moral disorientation, moral distress, moral injury, moral orienting system, moral stress, systemic assessment

## Abstract

Current conceptualizations of moral injury are limited by a reactive, symptom-based focus that risks pathologizing what are often systemic failures. This paper proposes a shift from a “triage model” to a proactive framework of Moral Orienting Systems (MOS). We define a moral orienting system as the dynamic stabilizing interplay of meaningful values, beliefs, behaviors, and relationships that shape moral identity. Drawing on chaplaincy experiences and interdisciplinary theory, we argue that moral wellbeing is not a static trait but a dynamic relation between an individual’s moral stress and the stability and strength of their moral orientation. When systemic strength is sufficient to metabolize stress, the result is moral affirmation; when overwhelmed, the result is moral disorientation. We contrast this framework with existing measures (e.g., MIES, MIOS) to highlight their limitations in capturing chronic, non-event-based moral erosion. Finally, we close by noting the need for a Moral Orienting System Assessment (MOSA) as an operational tool to map these vectors, offering a multi-actor case study to demonstrate how this framework might guide systemic intervention and moral reorientation.

## Introduction

The September 2025 update to the American Psychiatric Association’s DSM-5-TR, expanded the text for diagnostic code Z65.8 to “Moral, Religious, or Spiritual Problem” (1). While this classification existed previously, it only focused on explicitly religious or spiritual issues. The update formally recognizes moral suffering by explicitly adding “moral problems” and “moral injury” as clinically significant concerns, distinct from, though often co-occurring with, conditions like PTSD or depression. This increased clinical attention to the moral dimensions of human experience is a significant development that requires deliberate attention in application.

By framing moral suffering primarily through a diagnostic lens, there is a significant risk of pathologizing and individualizing what are often systemic, relational, and structural failures. This concern echoes and amplifies existing calls from scholars for a more nuanced and contextually-sensitive understanding that can account for the complex interplay of individual, social, cultural, and spiritual factors (2, 3). As Molendijk (2) argues, treating these states solely as individual pathologies places undue responsibility on the sufferer and obscures how moral emotions often function as valid indicators of organizational dysfunction rather than cognitive distortions. Our conceptual framework systematizes the context-informed approach Molendijk calls for.

We suggest that current conceptualizations and measurement approaches, while valuable, are limited by their fundamentally reactive posture. They function as a “triage model,” designed to identify harm after it occurs by focusing on individual symptoms and specific acute traumatic events. This paper proposes a necessary shift to a proactive and systemic framework. We argue that before we can accurately assess injury, we must first be able to map the “moral terrain” itself. Drawing on Moon’s theoretical development of “moral orienting systems” (4), we argue that before assessing “injury,” we must map the system’s overall status.

A moral orienting system is the stabilizing interplay of an individual’s meaningful values, beliefs, behaviors, relationships, and experiences that shape their worldview and decision-making. When a moral orienting system cannot accommodate or assimilate moral stress the individual experiences moral disorientation.

This new terminology allows us to create a clearer taxonomy: we reframe moral injury as a specific and acute form of moral disorientation (a “moral broken bone”). Conversely, the broader MOS framework is critically able to account for the chronic and systemic forms of moral suffering (something like “moral osteoporosis”) that current event-based approaches miss. Our primary contribution is twofold: theoretical and methodological.

Theoretically, we articulate the construct of “Moral Orienting Systems” and clarify that moral disorientation is a state of systemic incoherence within the MOS. We show why this system-level lens captures chronic, relational dynamics that event-focused accounts miss. Methodologically, we build the case that this systemic lens requires a new assessment approach to address the limitations of current tools. To show this framework’s practical traction, we include a brief multi-actor case that maps interacting moral orienting systems and illustrates how such mapping can guide care and organizational action. This argument logically culminates in the need for such a tool, and thus we close by noting the ongoing development of a companion instrument, the Moral Orienting Systems Assessment (MOSA), which is being designed to operationalize this framework.

## Review of the literature: conceptualizations and measurement scales

The concept of moral injury was first introduced by Shay (1994) to describe the psychological and spiritual wounds of combat veterans who had experienced a betrayal of “what’s right” by those in power. Shay argued that this moral betrayal could shatter a service member’s trust in self, others, and the world, leading to a profound sense of disorientation and disconnection. Litz and colleagues (5) expanded on this concept, defining moral injury as the lasting impact of “perpetrating, failing to prevent, bearing witness to, or learning about acts that transgress deeply held moral beliefs and expectations” (p. 697). They proposed a preliminary model of moral injury that emphasized the role of self-blame, guilt, and shame in mediating the relationship between morally injurious events and adverse outcomes such as PTSD, depression, and suicidality.

Since these seminal works, research on moral injury has grown rapidly, with numerous studies documenting its prevalence and impact among military populations (6; Currier et al., 2015; 7, 8). This research has led to the development of several self-report measures of moral injury. This history of measurement is not random; it reflects a clear conceptual evolution in how the field has understood moral suffering. This progression can be understood as navigating a persistent methodological tension between “psychometric precision” and “conceptual breadth.” Early instruments, representing the high-precision pole, were developed for narrow, specific populations. Later instruments aimed for greater breadth to become applicable to diverse populations, often by focusing on symptoms. Critically, as we will argue, this entire evolution has largely remained within an individualistic paradigm, necessitating the systemic framework we propose.

The MIES is the first validated measure of moral injury, developed and published in 2013 by Nash et al. for use in military populations. It is a 9-item scale that assesses perceived transgressions by self or others (e.g., “I saw things that were morally wrong”) and perceived betrayals (e.g., “I feel betrayed by leaders who I once trusted”) in a military context. Items are rated on a 6-point Likert scale from “strongly disagree” to “strongly agree,” yielding a total score and two subscale scores (transgressions and betrayals). The MIES has demonstrated good internal consistency, test-retest reliability, and convergent validity with measures of PTSD, depression, and suicidality in military samples (6, 9).

The MIQ-M is a 20-item scale that assesses both the frequency and impact of morally injurious events, using separate stems for each item (e.g., “I did things that betrayed my personal values” and “How much distress does this currently cause you?”). Items are rated on a 4-point Likert scale from “never” to “often” for frequency and “none” to “severe” for impact, yielding a total score and four subscale scores (moral violations by self, moral violations by others, betrayal by others, and moral struggles). The MIQ-M has shown good internal consistency and convergent validity with measures of PTSD, depression, and suicidality in military and veteran samples (Currier et al., 2013; Currier, Holland, Rojas-Flores et al., 2015).

The MISS-M is a 45-item scale that assesses ten dimensions of moral injury outcomes, including guilt, shame, betrayal, moral concerns, loss of meaning, difficulty forgiving, self-condemnation, spiritual/religious struggles, and loss of trust. Items are rated on a 5-point Likert scale from “strongly disagree” to “strongly agree,” yielding a total score and ten subscale scores. The MISS-M has demonstrated excellent internal consistency, test-retest reliability, and convergent validity with measures of PTSD, depression, anxiety, and quality of life in military and veteran samples (10).

Another similar instrument is the Expressions of Moral Injury Scale-Military Version (EMIS-M). Developed by Currier and colleagues (2018), this scale assesses the behavioral, social, and spiritual expressions of MI. It has strong psychometric properties and usefully differentiates between self-directed and other-directed expressions of blame. However, from a systemic perspective, it remains limited. By focusing on an individual’s “expressions” of injury, it maintains an individualistic paradigm, paying little attention to the systemic context that caused those expressions in the first place.

The Moral Injury Outcome Scale (MIOS) is a more recently developed 14-item scale that assesses the impact of moral injury experiences. It was created through a multinational qualitative analysis of interviews with service members, veterans, and clinicians, followed by exploratory and confirmatory factor analyses and cross-national invariance testing. The MIOS consists of two subscales: Shame-Related (SR) outcomes and Trust Violation-Related (TVR) outcomes. Respondents are asked to rate the extent to which they agree with each item in relation to their worst and most currently distressing potentially morally injurious event (PMIE) in the past month. The MIOS has demonstrated strong internal consistency, a robust two-factor structure, and partial scalar invariance across nations. It provides a concise, psychometrically sound measure of key moral injury symptoms that is linked to a specific PMIE, allowing clinicians to target the impact of a particular morally injurious memory and track changes over time (11).

Recent developments have been characterized by the expansion of the MI construct beyond the military, leading to a rapid proliferation of new instruments since 2023 designed for civilian and general populations. The Moral Injury and Distress Scale (MIDS), for example, represents a significant advance as a cross-population tool validated for veterans, healthcare workers, and first responders (12). It integrates PMIE exposure (commission, omission, witnessing) with 18 outcome items and provides a validated clinical cut-score for clinical use. This wave also includes the Occupational Moral Injury Scale (OMIS), designed for any occupational setting by linking work-specific events to markers like guilt and loss of trust (13), as well as the Adult Moral Injury Scale (AMIS), a 39-item measure for the general population that specifically assesses spiritual and existential crises (14). Alongside these broad measures are highly specific adaptations, such as the 10-item Moral Injury Symptom Scale - Clinician Version Short Form (MISS-CV-SF), developed in 2024 specifically for acute care nurses (15).

While these measures have made valuable contributions to the assessment and study of moral injury, they are hampered by the very limitations this paper seeks to address. As we argued in our introduction, these tools are 1) fundamentally event-focused, failing to capture the broader structural context of a moral worldview. They 2) frame moral injury as individual pathology, focusing on symptoms like guilt and shame while overlooking the larger existential and relational dimensions. Finally, 3) they remain context-bound, with language and content heavily grounded in military norms that are less applicable to diverse moral landscapes. These concerns echo findings from a comprehensive review of MI literature, which highlighted inconsistent definitions, the lack of a gold-standard outcome measure, and a predominant focus on military contexts as significant limitations hindering the field’s advancement (16). **Table 1** summarizes these existing approaches and highlights how the MOS framework addresses these specific systemic gaps.

**Table 1:** Comparative analysis of existing moral injury measures vs. the Moral Orienting Systems (MOS) framework.

Instrument/Approach	Primary focus (Unit of analysis)	Key strength	Systemic limitations (The “Gap”)	How MOS/MOSA extends this
MIES/MIQ-M (9; Currier et al., 2015)	Symptom frequency & perceived betrayal. *(e.g., “I saw things that were morally wrong”)*	Validated for military populations; identifies specific index events and betrayal types.	Event-Bound: Requires a specific transgression to score. Individual: Focuses primarily on intrapsychic symptoms (guilt/shame) rather than systemic context.	System-Bound: Assesses the *coherence* of the moral system itself (values, relationships), regardless of whether a specific “event” has been identified.
MIOS (11)	Impact of a specific “Index Event” (PMIE). *(e.g., Shame-Related & Trust-Violation outcomes)*	High internal validity; differentiates between Trust and Shame outcomes; widely used internationally.	Reactive: Functions as a “triage” tool after injury occurs. Acute Focus: Misses chronic, erosive moral stress (“moral osteoporosis”) that accumulates without a single PMIE.	Proactive: Maps the stability of values and relationships *before* or *during* stress, capturing chronic disorientation rather than just acute injury.
MIDS (12)	Cross-population exposure & distress. *(Healthcare, First Responders, Veterans)*	Context-sensitive; captures diverse exposure types (omission, witnessing); validated clinical cut-scores.	Pathology-Focused: Still prioritizes distress scores over systemic health. Deficit Model: Measures what is *wrong* (symptoms) rather than what is *missing* (stabilizing factors).	Strengths-Based: Assesses stabilizing factors (meaningful relationships, value clarity) alongside distress, identifying resilience resources within the system.
MOS Framework (Proposed)	Systemic Interplay *(Values, Beliefs, Behaviors, Relationships)*	Ecological: Maps the “terrain” and relational web, not just the injury. Captures the “stabilizing” function of the system.	*N/A (Theoretical Framework)*	Operationalization: Targets the “inability to accommodate” moral stress rather than just symptom severity, allowing for preventative mapping.

In recent years, the moral injury construct has begun to be applied beyond military and veteran populations to a variety of other professions and life contexts where individuals may encounter situations that profoundly violate their moral foundations. This expansion has been driven by the recognition that many challenging human experiences can give rise to a form of moral suffering. This has been documented everywhere from frontline medical work to international humanitarian efforts, and urban primary school education (17, 18).

In healthcare settings, for example, the moral stress and anguish experienced by providers during events like triage decisions, medical errors, or ethical violations has been conceptualized as a manifestation of moral injury (19, 20). Among humanitarian aid workers, researchers have explored how chronic exposures to human suffering, bearing witness to atrocities, and being unable to provide adequate relief can inflict moral-existential wounds (21). Teachers and school personnel may develop moral injuries from being compelled to engage in or allow disciplinary practices that violate their values about child wellbeing (22). Social workers have reported profound moral distress from having to make decisions that compromise the safety and wellbeing of children, such as separating families or returning children to abusive homes due to systemic pressures (23). Even parents dealing with child protective services have described experiences evocative of moral injury, such as having to comply with requirements that seem antithetical to their children’s needs, or witnessing their children suffer harm while under state care (23).

In studying moral injury across these diverse contexts, researchers have often adapted the existing military-focused instruments like the MIES, MIQ-M, and MISS-M by modifying certain items to be more applicable or relevant to the given population (e.g., changing “combat” to “work” or removing references to the military). However, much of this broadening work has maintained a predominantly intrapsychic and symptom-focused orientation inherited from the original military constructs. The emphasis remains on assessing individuals’ exposures to potentially morally injurious events and their ensuing psychological reactions like guilt, shame, anger, and spiritual struggles. In contrast, the MIDS was explicitly designed for diverse civilian populations and avoids superficial item substitution by embedding context-sensitive exposure types (e.g., omission, witnessing) and eliciting narrative reflections that allow moral injuries to be interpreted within broader relational and institutional frameworks. While an excellent intervention, it still largely maintains an individualistic paradigm that conceptually narrows the moral injury phenomenon.

Moral injury has already been shown to arise from ruptures between persons and the shared moral value spheres that help constitute their identities and lived worlds (24). Morality is inherently intersubjective, shaped by our relationships, communities, institutions, and cultural traditions, which imbue our lives with norms, ideals, and moral demands (Mattingly, 2014). When we perpetrate, witness, or are subjected to acts that transgress these moral horizons, the injury extends beyond the individual psyche to a rupturing of our felt moral belonging and recognition (25; Wilde, 2022). We argue that at its core, what we understand as “moral injury” can be seen as dynamic processes unfolding within specific moral-relational worlds, whose psychological and existential wounds cannot be fully grasped solely at the level of individual minds and symptoms. While the moral injury construct remains valuable for understanding acute, event-specific transgressions, a broader conceptual framework is necessary to capture more diffuse, systemic, and relational dynamics.

Moral injury can disrupt one’s basic trust in moral values and the social-moral orders that encode them. It can shatter assumptions about responsibility, justice, and the moral character of individuals, communities and institutions we once relied upon (17). At a core existential level, it can leave persons adrift, unable to make meaning of their identities and experiences through the available moral-cultural frameworks. The profound distress of moral injury stems not just from exposure to aberrant events, but from how those events reverberate through and potentially erode one’s entire moral world and way of being (26).

This intersubjective view highlights how moral injury is always irreducibly embedded within webs of relationships, traditions, social-institutional forces, and systems of power that give rise to particular moral valences and violations in the first place. A survivor of genocide may develop moral injury, but that injury will be shaped by cultural, historical, and political realities that enabled such an atrocity. A healthcare provider’s moral injury will reflect professional and organizational norms around ethical practice. Violations against a marginalized group stem from and feed back into broader structures of inequality and injustice over generations.

These transgressions must be understood as dynamic processes unfolding within specific moral-relational worlds that are continually shaping and being shaped by persons’ beliefs, values, and ways of being (4; Wilde, 2019). More holistic, context-attentive approaches are needed to characterize the intersubjective textures and reverberations of moral injury and repair across diverse moral ecologies. This intersubjective critique reveals a significant limitation: existing conceptual frames and measurement tools do not adequately capture the relational, cultural, and moral worlds within which moral injuries take form and inner meanings accrue. The MIES, MIQ-M, MISS-M and other adaptations focus primarily on individual appraisals of transgressive events and psychological-spiritual reactions like guilt, shame and meaning loss. While important, these measures do not grapple with the broader moral-relational matrices that shape how persons experience and make sense of morally injurious events.

Recently, Vasic and Dorais have suggested that “Relational-Cultural Theory” might be a useful framework for considering moral injury given similarities in theoretical foundations (27). Their work does take into account more intersubjective and interpersonal dimensions, however it continues to proceed with the presumption that moral injuries are primarily to be considered acute. These certainly occur, but we are also interested in exploring the consequences of persistent structural harm. A broken bone because of a fall is worth treating, but so is a marked decline in bone density as a result of daily exposure to heavy metals like lead and cadmium. To more comprehensively assess moral injury, we need measures that can characterize the intersubjective moral landscapes people inhabit: their foundational moral values and identity commitments, the moral traditions and communities that are sources of moral meaning, the moral power dynamics and structural injustices that give rise to particular moral violations in their lives.

We suggest there is a need for an assessment that explores narrative sensemaking processes around morally injurious events in a way that better accounts for intersubjectivity, relationality, and the structural dimensions of moral stress. This can lead to a better understanding of how moral injuries can disrupt moral self-understandings, damage moral relationships, and rupture senses of moral agency and belonging. Following from this, we need ways to understand processes of moral repair, reconstructing moral identities, reclaiming moral voices and positions, and efforts to reform damaged moral systems that enabled the injuries.

This critique reveals a clear and significant gap in the field: a need for new assessment tools capable of capturing the systemic, relational, and chronic nature of moral suffering that current event-based instruments miss. The following sections build the theoretical framework for such a tool, based on a shift to a “Moral Orienting Systems” framework, which we believe can accomplish this.

## Reconceptualizing moral injury as moral disorientation

Scholars like Molendijk (2) have recently argued that the field must move beyond individual pathology to adopt “context-informed” approaches that treat moral injury as a systemic signal rather than a mere symptom. Responding to this theoretical necessity requires a new architecture. In *Warriors Between Worlds*, Zachary Moon (4) offers a compelling framework that addresses this need. This model was not developed in the abstract but emerged directly from extensive chaplaincy work with military service members and veterans. In these contexts, standard symptom-based interventions often failed to resonate with the depth and complexity of service members’ experience; they reported not just acute distress from specific events, but a pervasive sense of being “lost”—an inability to locate themselves within their previously held moral maps. Consequently, Moon argues that moral injury is best understood not as a set of discrete symptoms, but as a fundamental disruption in the architecture of the moral orienting system itself.

If the definition provided in our introduction describes the function of this system (stabilization), Moon’s work elucidates its nature. He describes the MOS not as a static entity, but as an “existential and embodied reality” (p. 6) that grounds a person’s sense of self, purpose, and belonging within a larger moral and cultural matrix. It is a fluid and contextually-embedded process of meaning-making that evolves over the lifespan. As noted, one’s MOS is capable of accommodating or assimilating new experiences; when that capacity is overwhelmed, the result is the systemic disruption we term moral disorientation. Consequently, we understand one’s moral orienting system status not as a static trait or unidimensional concept, but as a dynamic relation between two primary vectors: the intensity of moral stress encountered and the strength of the system available to sustain it. To understand the specific nature of this disruption, it is helpful to contrast it with the well-established concept of “moral distress.”

Using a navigational metaphor, if moral disorientation is the loss of one’s map or compass, moral distress is better understood as a blocked path. Moral distress typically arises “when one knows the right thing to do, but institutional constraints make it nearly impossible to pursue the right course of action,” (28, p. 6). While subsequent definitions have debated whether a clear moral judgment is strictly necessary or if moral uncertainty or dilemmas can also precipitate it, the core often involves psychological distress stemming from the inability to act according to one’s moral commitments due to constraints, which may be external (like institutional policy) or internal (like fear or self-doubt). Morley and colleagues (29) synthesized the literature to suggest the necessary and sufficient conditions for moral distress are the experience of a moral event, subsequent psychological distress, and a direct causal link between the two. Moral disorientation, framed through the MOS lens, represents a more fundamental and systemic phenomenon.

While it *can* be triggered by events involving constrained action, it encompasses a broader disruption of the entire orienting system, the fracturing of meaningful linkages to values, beliefs, behaviors, *and* relationships. From a systemic perspective, this disruption arises when the moral stress intensity overwhelms the strength of the system, destabilizing one’s core sense of identity, meaning, purpose, and belonging. This could be from acute events, chronic exposure to morally corrosive environments, or the loss of the stabilizing dimensions of support. We conceptualize this as a dual-system dynamic, where moral orientation is defined by the interaction between these two distinct vectors: the moral stress intensity impacting the individual and the strength of the system available to sustain them.

Critically, this model does not assume that moral stress is inherently negative. When an individual’s strength of system is sufficient to metabolize the stress intensity, the result is not disorientation but moral affirmation, a strengthening of the moral core through the successful integration of challenge. Disorientation only occurs when the stress overwhelms the system’s capacity to accommodate it. Where moral distress often highlights a conflict between judgment and action in a specific context, moral disorientation signifies a deeper unraveling or loss of coherence *within* the moral framework itself, leaving the individual feeling morally adrift.

Framed this way, “moral injury” is generated by a severe disruption to one’s moral orienting system, including experiences of transgression, betrayal, or moral ambiguity. These experiences can challenge core assumptions about oneself, others, and the world, leading to a sense of moral confusion, alienation, and despair. Moral injury, then, is not just a matter of individual symptoms, but a rupture in one’s social-relational world and capacity for life-giving meaning-making.

Moon identifies several key dimensions of moral orienting systems that can be disrupted by morally injurious experiences:

1. Beliefs and values: The fundamental assumptions, principles, and commitments that define one’s sense of right and wrong, good and evil, just and unjust. Morally injurious experiences can shatter these beliefs and values, leading to a sense of moral confusion, relativism, or nihilism.
2. Behaviors and actions: The concrete ways in which one enacts, practices, or embodies one’s moral beliefs and values in the world. These include routines and habits, some may appear mundane and insignificant, but have importance in connecting one to their social-relational world (e.g. work routines, family gatherings, participation in faith community practices or worship, etc.) Morally injurious experiences can create a profound sense of dissonance or disconnection impacting their sense of moral agency, leading to feelings of guilt, shame, and self-condemnation.
3. Relationships and communities: The social contexts and bonds that shape and sustain one’s moral identity and sense of belonging. Morally injurious experiences can rupture these meaningful relationships, leading to a sense of isolation, distrust, and exile.
4. Meaning and purpose: The overarching narrative or framework that gives coherence, direction, and significance to one’s moral life. Morally injurious experiences can shatter this sense of meaning and/or goodness, leading to feelings of futility, hopelessness, and existential crisis.

Moon argues that these dimensions of moral experience are not independent but deeply interconnected and mutually reinforcing. A disruption in one area can reverberate throughout the entire system, creating a cascading crisis of moral identity and meaning. For example, a soldier may not only experience intense guilt and shame (moral emotions), but also a shattering of core beliefs about the righteousness of the mission (moral beliefs), a sense of betrayal by commanding officers (moral relationships), and a loss of faith in the ultimate meaning and purpose of the war (moral meaning). To situate this Moral Orienting System framework in relation to the concepts of moral distress and moral injury discussed earlier, **Table 2** offers a comparative taxonomy.

**Table 2:** Comparative taxonomy of moral distress, moral injury, and moral disorientation.

Construct	Core definition	Unit of analysis	Primary metaphor
Moral Distress	Knowing the right action but being constrained from performing it.	Individual Constraint	A Blocked Path
Moral Injury	Lasting impact of transgressing core moral beliefs, often from an acute event.	Acute Transgressive Event	A Broken Bone/Wound
Moral Orienting System	The “moral terrain” itself; the interconnected system of one’s core beliefs, key relationships, and sense of purpose.	The Systemic Coherence/The Relational Web	The Moral Terrain/The Map
Moral Disorientation	A persistent disruption or loss of coherence within one’s Moral Orienting System (MOS).	The State of the System/Systemic Disruption	A Lost Compass (on the terrain)

This focus on the entire Moral Orienting System, as defined in **Table 2**, is a key component of the theory. It explains why the resulting moral disorientation cannot be reduced to a set of individual symptoms or pathologies (the “broken bone” model) but represents a profound existential and relational rupture that requires a holistic and contextually-sensitive approach to healing and repair. Moon envisions this process as a “moral reorientation” that involves not only alleviating individual symptoms linked to discrete events but repairing the entire dynamic system (beliefs, values, behaviors, relationships) within its social context. It may involve a range of interventions and practices, such as:

1. Narrative reconstruction: The process of telling and re-telling one’s story to integrate the experience into a coherent narrative of self and world. Unlike event-focused approaches, this explicitly maps shifts in beliefs, relational trust, and behavioral patterns over time to reconstruct the self within its disrupted moral landscape.
2. Moral dialogue and disclosure: Sharing one’s story with empathic others to move beyond individual unburdening toward intersubjective meaning-making. This process is crucial for repairing ruptured bonds and co-constructing new moral understandings within the communities or institutions involved.
3. Moral reflection and education: The critical examination and refinement of one’s moral beliefs and assumptions. This extends individual reflection to include an analysis of the systemic context—such as institutional norms and pressures—that contributed to the disorientation.
4. Moral action and atonement: Taking concrete steps to make amends, seek forgiveness, or engage in restorative justice. This shifts the focus from individual reparation to actions aimed at rebuilding trust within the relevant system and addressing the systemic conditions that led to the disruption.
5. Moral re-engagement and belonging: The re-establishment of meaningful relationships and roles within social-moral communities. This involves actively finding or co-creating spaces where the individual’s reconstructed moral identity can be lived out and affirmed.

These five processes are the practical methods for achieving moral reorientation, functioning to systematically rebuild the core components of a healthy moral orienting system. This reparative work overlays the individual with the collective viewpoint, shifting attention to underlying meaning structures that exist between individuals and society. Following Buechner, we suggest that an emphasis on this intersubjective repair and reknitting of connection can be “a first step toward imagining and enacting other possibilities or enlarging the moral imagination” (2020). Moon’s framework proposes that this expansion and reconnection is a result of the equilibrium between life-giving and life-limiting experiences.

**Figure 1:**
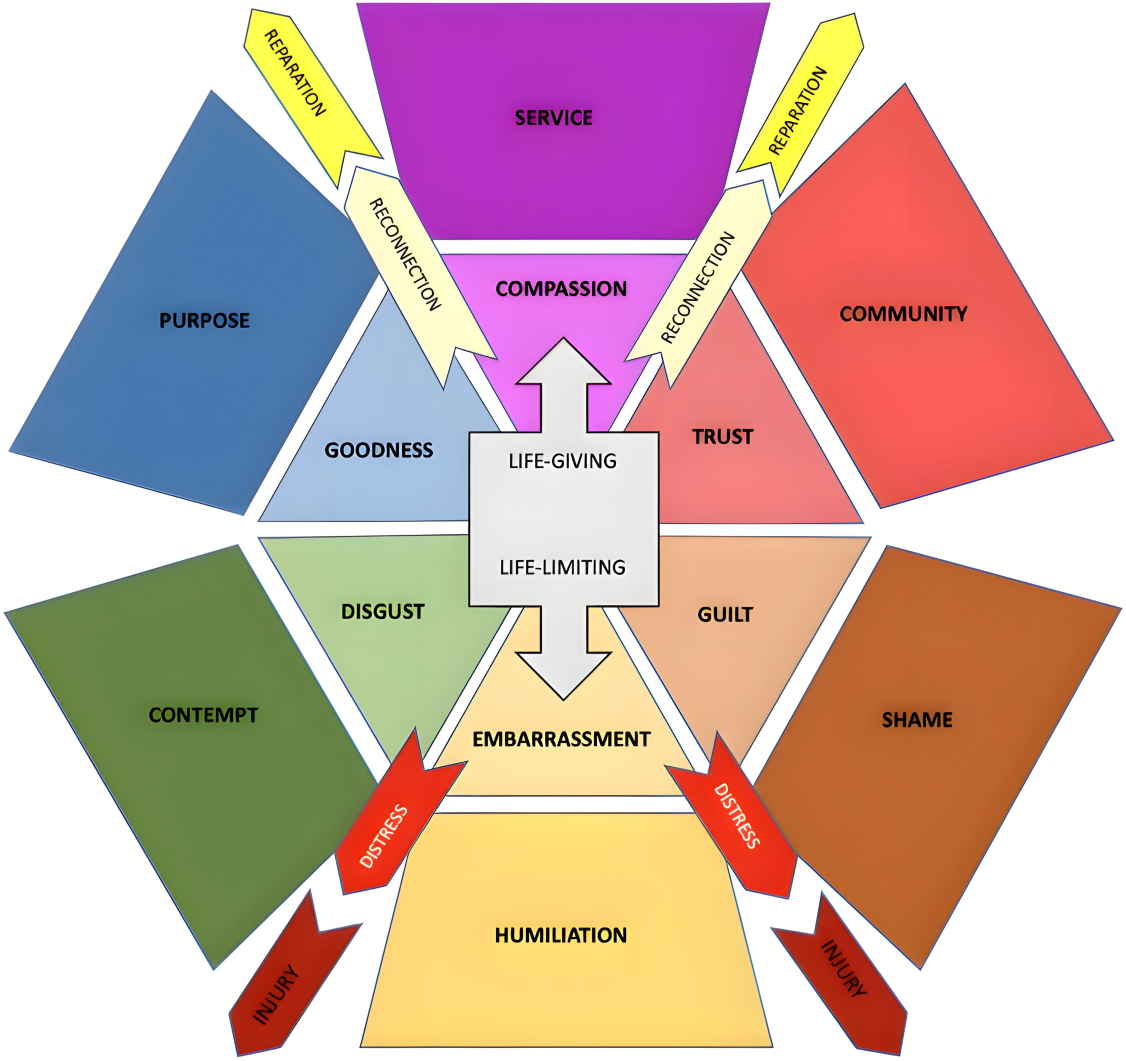
Diagram of life-giving and life-limiting emotional experiences within the moral orienting system.

This intersubjective, holistic perspective aims to restore the life-giving existential states of trust, purpose, and community that moral disorientation erodes, while mitigating the life-limiting states of shame, humiliation, and contempt. It is visually captured in **Figure 1** below, which maps the emotional experiences that can either enhance one’s moral groundedness (the “life-giving” experiences of service, compassion, trust) or undermine it (the “life-limiting” states of humiliation, shame, contempt). As the diagram illustrates, life-giving experiences like recognizing the humanity of others through compassion, or finding purpose through trusting relationships and community engagement, can strengthen an individual’s moral orienting system. Conversely, injurious experiences that elicit self-condemning emotions like guilt, embarrassment, and shame can initiate an unraveling of one’s ethical moorings, moral self-concept, and sense of existential meaning.

This conceptual model provides a framework for understanding how potentially morally injurious experiences can disrupt the coherence and integration of an individual’s entire matrix of moral beliefs, behaviors, relationships, emotions, and meaning-making capacities. The circular, interconnected nature of the diagram underscores how states of moral orientation and disorientation are not static or siloed, but dynamically shaped by the accrual and reverberation of life experiences across multiple domains. An initial experience of humiliation, for instance, can precipitate a cascade of contempt, disgust, and relational injury that compounds the disruption of one’s moral orienting system. Healing thus requires a holistic process of moral reorientation to reconstruct an integrated and life-enhancing system of ethical identity, grounding, and connectedness.

Moral orienting systems encompass the dynamic interplay of an individual’s beliefs, values, behaviors, relationships, emotions, and sense of meaning and purpose, contextualizing them relationally and intersubjectively with others. These systems are shaped by a complex array of biological, psychological, social, cultural, and spiritual factors, and provide a fundamental framework for navigating the ethical challenges and ambiguities of human experience. When an individual’s moral orienting system is functioning optimally, it provides a cohesive and resilient framework for maintaining moral integrity, meaningful relationships, and a sense of existential grounding in the face of life’s complexities.

When an individual is exposed to potentially morally injurious experiences that profoundly violate or betray their core moral beliefs and expectations, the resulting moral anguish can precipitate a state of moral disorientation that reverberates throughout their entire moral orienting system. This disorientation is characterized by a pervasive sense of moral confusion, alienation, and existential crisis that undermines the coherence and stability of an individual’s moral worldview, identity, and relationships. This systemic disorientation is more foundational than the acute symptom clusters often described as moral injury, as it represents a disruption to the underlying architecture, the dynamic interplay of meaningful values, beliefs, behaviors, and relationships that structure moral meaning-making, identity, and relationality itself.

Moral injury, within the MOS framework, can be understood as a particular type or manifestation of severe moral disorientation that arises from specific morally injurious events or experiences, such as perpetrating, failing to prevent, or bearing witness to acts that transgress deeply held moral beliefs and expectations. However, while moral injury represents a distinct and particularly severe form of moral disorientation, it is not the only way in which an individual’s moral orienting system can be disrupted or challenged. Other experiences, such as chronic exposure to morally ambiguous or ethically complex situations, can also contribute to a more gradual erosion of moral meaning and coherence over time (Sugrue, 2024; 30, 31). Similarly, systemic experiences of oppression, injustice, or marginalization can create a profound sense of moral disorientation by undermining an individual’s basic trust in the fairness and decency of the social world.

By situating moral injury within the broader framework of moral disorientation, we can develop a more comprehensive and nuanced understanding of the nature and impact of moral suffering, as well as the pathways toward moral reorientation. This expanded perspective has important implications for research, clinical practice, and social advocacy, as it calls for a more holistic and systemically-oriented approach to addressing the complex and multidimensional nature of moral anguish. As we conceive of it, the disorienting experience involves multiple interrelated dimensions:

- Cognitive Disorientation: Shattered assumptions about moral order, justice, humanity’s capacity for good/evil. Inability to make sense of experiences that defy previous belief systems.
- Emotional Disorientation: Overwhelming moral emotions like disgust, contempt, self-condemning shame that become entrenched and distort self-perception. Affective detachment from previous sources of moral worth.
- Relational Disorientation: Ruptures in attachments to social-moral communities that previously anchored one’s values and identity (e.g. professional ethics, faith traditions, cultural worldviews). Alienation from those who cannot understand the depth of moral anguish experienced.
- Existential Disorientation: Loss of orienting sense of purpose, meaning, and philosophical grounding. Feelings of being adrift in the moral universe without guiding ethical/spiritual compass.
- Somatic/Behavioral Disorientation: Breakdown of value-congruent patterns of behavior and embodied ethical know-how. Visceral experiences of moral incoherence and self-betrayal.

Functionally, we use “moral disorientation” to name what happens when an individual’s moral orienting system loses coherence over time. Rather than a list of symptoms or a single traumatic moment, moral disorientation is a system-level disruption of moral bearings, trust, identity, and/or agency. People describe not simply feeling hurt or constrained, but feeling unmoored, unsure which norms apply, whom to trust, and who they are as a moral actor in their roles. This can be precipitated by transgressions or betrayals, but it can also accumulate through grinding organizational contradictions and value conflicts. Framed this way, moral disorientation complements event-focused accounts of moral injury and constraint-focused accounts of moral distress by diagnosing the broader loss of orientation that makes judgment and aligned action harder to sustain.

As a conceptual framework, the term “moral disorientation” has several advantages over “moral injury,” especially for understanding moral suffering that might be comparatively milder than a single severe tragic event yet is routine and continuously taxes the moral orienting system. In situations such as these we feel the MOS is a better-fitting theoretical construct to use as a basis for assessment. We see at least four reasons why this is the case.

First, it accommodates a wider range of morally destabilizing experiences beyond discrete traumatic events, including ongoing systemic injustices, ethically toxic environments, and prolonged navigations of conflicting moral demands. Its dynamic, systemic emphasis captures how individuals may feel morally adrift not due to a singular injury, but the erosive impacts of challenging conditions on their moral orienting systems.

Second, moral disorientation inherently invokes the individual’s psychosocial ecology and cultural/structural contexts as pivotal forces shaping moral experience. Rather than reducing moral suffering to intrapsychic breakdowns, it calls attention to the societal, organizational, and relational matrices in which our moral orienting systems are embedded and either nurtured or eroded over time.

Third, moral disorientation provides a more inclusive framework for understanding the textures of moral anguish across diverse occupational and community contexts where ethical challenges arise, from humanitarian aid work to institutional leadership to dehumanizing experiences of structural oppression. The language of moral disorientation validates the human struggle to sustain an integrated ethical identity in the world.

Finally, the MOS framework shifts the model from reactive to proactive and preventative. The “moral injury” construct is a triage model; its tools are used after an acute, morally injurious event has caused harm, much like responding to a broken bone. In contrast, the MOS framework functions as a diagnostic tool to assess systemic health before a crisis. This allows for a more holistic approach interested in cultivating moral well-being and strengthening systemic integrity, not just responding to “injury.”

## The MOS frame in use: a case study with analysis

To illustrate the practical utility of the Moral Orienting Systems (MOS) framework in analyzing complex moral situations beyond acute, event-specific injury, we present the following composite case study. This narrative integrates common themes and dynamics observed in various professional and institutional contexts where moral disorientation can arise. Following the case description, we offer an analysis that maps the disruptions and interplay within the moral orienting systems of the key individuals involved, demonstrating how the MOS lens illuminates the relational, systemic, and existential dimensions of moral suffering and potential pathways toward reorientation.

### Case vignette

On most days, Rev. Dr. Isabel Chen’s office at Northtonshire University smells faintly of peppermint tea. Students had learned that if they crossed the threshold of her door and sank into the mismatched armchairs, the pace of the campus slowed for a while. They also learned that what was said there stayed there, save for the few, clearly explained exceptions that honored both the law and dignity.

The rain that Tuesday made the campus shine like a polished stone. Signs for the new capital campaign were everywhere, but the rain was soaking everything. Daniel came in drenched, a first-generation student who still knocked even though she’d told him not to. He sat in the armchair and started chatty, but Isabel could tell something was wrong. Eventually, he quieted. “Is there anything else I wonder, Daniel?” Isabel asked.

In response Daniel began to tell a story that tumbled forward in stops and starts. He told bits out of order and it was hard to follow. About scholarship obligations, about donor “opportunities,” and how Daniel was part of the student team that wowed donors. A story about a trustee everyone called Mr. Harrison, who told jokes that were not jokes. Daniel didn’t like it but felt so grateful for the financial support of the school that he said he thought he could swallow it. But yesterday at an event Mr. Harrison started to give a back rub that wasn’t asked for. One that wasn’t right. Daniel grew quiet. “You can’t say anything Dr. Chen. Please. With all that’s going on I can’t…. I … I just don’t know what to do.”

Isabel named what she heard, mirrored his language back with clarity but without heat. Then she moved further. “Am I hearing it right that you’re grateful, but feeling afraid and your body told you this isn’t safe?” He nodded. “All right,” she continued, steady. “That sounds like something we should address. Would you like me to walk with you to the Dean of Students so you can make a report?” A beat, then a slow nod. Together they went to the Dean and Daniel shared his story.

The next week moved too slowly. Daniel didn’t come out to several chaplaincy events and none of the other chaplains said they’d heard from him. Isabel wrote a couple emails to no response. Eventually she texted him and said she’d love to touch base if he was willing. The next afternoon Daniel came back, eyes tired, shoulders pitched forward. Eventually it came out: “Nothing’s happened,” he said. Then he told a story about a meeting with an associate dean who used words like “sensitive” and “learning to navigate powerful personalities.” He repeated the phrase “long-time friend of the university.” Isabel felt anger rise behind her ribs. She tried to support Daniel as best she could, and when the meeting was over she scheduled a meeting with John Darwell, a senior administrator she trusted.

She laid out the facts and asked what was going on. Before even a quarter of the story was out, John stood and the office door clicked shut. When he returned he asked her to finish. When she did he replied carefully, his voice dropping. “The situation,” he said, “is delicate.” He said it was being handled with discretion. He said Mr. Harrison’s financial pledge was the cornerstone of the entire University-wide campaign. Then he said the part she would remember word-for-word months later: As chaplain, could she help Daniel build “professional resilience”? Could she equip him spiritually to bear the complexities of donor relations? “We’ll make sure they’re never together again, but … is there any other way to manage this?”

Isabel’s first sensation was not outrage but vertigo. The room tilted. She had escorted a student into a system that asked him to be small so the gift could be large. She realized that the confidence she had in the university had been collateral in the campaign’s public story. Now she felt like she was being asked to lay a blessing on suffering. “John, I don’t believe what I’m hearing you say.” John hung his head some. “Life is complicated, Bel. We’ll figure this out.” But Isabel was not at all sure that “figuring it out” would be what Daniel needed.

### MOS mapping of case

A traditional, event-focused moral injury model would likely interpret this case by focusing exclusively on Daniel as the “injured” party. It would identify the Potentially Morally Injurious Event (PMIE) as the university’s betrayal of trust and its failure to act on his report. The analysis would then center on assessing Daniel’s individual psychological symptoms: his withdrawal from chaplaincy events, his anger, and his distrust. Consequently, the intervention would likely be individual-focused therapy for Daniel. This traditional lens, however, would almost entirely miss Isabel’s moral disorientation and the slow, corrosive erosion of John’s own moral coherence as he becomes complicit in the institution’s “moral drift.”

In contrast, the Moral Orienting Systems framework provides the opportunity to assess this complex situation as a systemic moral ecology, revealing the relational disorientation the event-focused model overlooks. As Keefe-Perry describes well in *Tending Call*, “When [meaningful] communities no longer feel safe, or when people experience alienation from their mentors, peers, or moral communities … These relational disruptions can erode a person’s sense of meaning and purpose, leaving them adrift, unsure whether what once gave their life coherence still holds” (32, p. 21). An MOS perspective identifies each subject in the case study as a moral subject embedded in a context marked by meaningful beliefs, values, behaviors, and relationships that organize their participation on the university campus and support our understanding of how each of them is adversely impacted in this situation.

The MOS frame claims that morality is not an individual possession; it is co-constructed and sustained within relationships, communities, and institutions. Most constructs and measurement scales fail to account for how power dynamics, institutional betrayal, and structural injustices create the conditions for moral disorientation in the first place. The case study vividly illustrates this point: the injury is not located solely within Daniel’s psyche, but within the university’s system of values that seems to functionally prioritize donations over student safety. Each of the actors in the case is embedded in this context to different effect.

Isabel’s moral orienting system is deeply involved in how she navigates this situation. Her beliefs and values (dignity, safety, accountability, integrity, care) guide her deliberate actions. Her meaningful relationships across multiple communities is influential in her sense of alignment and transgression at different times. Her sense of meaningful relationship with students, including Daniel, motivates her leadership and sense of ethical action, while her meaningful relationship with John and university institution more broadly plays a role in how she experiences moral disorientation. When these meaningful beliefs, values, and relationships are put into conflict with Daniel asking for her to advocate for his justice and the university asking her to pacify Daniel’s suffering, she experiences moral disorientation, reflecting the “vertigo” that signals a systemic rupture across her MOS.

Daniel does not inhabit the university setting like other students, his status as a first generation college student deeply shapes his experience. That he has become a featured token in donor-facing initiatives means in addition to pressure he may feel from his family and home communities, the university institution is also applying certain pressures on Daniel to “represent.” With this pressure comes a sense of responsibility which may be felt in life-giving ways like gratitude, loyalty, and purpose, but may also haunt him as self-doubt, as in the “imposter syndrome,” embarrassment, or guilt. These conditions influence how Daniel navigates the way his safety is endangered, his choices to risk disclosure and reporting, and then his withdrawal when reparative actions are not taken by those with institutional power.

Daniel’s meaningful relationships are crucially important in this situation: the strain this event may place on Daniel’s sense of himself and his relationships with family and friends outside the university, and then his exercising of trust and connection in his meaningful relationship with the campus chaplain as well as the university community and systems as a whole. The support and honoring that Daniel may have felt previously are called into question, if not outright betrayed, with anger, despair, and distrust possible signature moral emotions. This incident exposes the moral labor that Daniel carries in this context, bearing the responsibility of choices not his own to protect the safety of the university above his own safety. Behaviorally, Daniel’s withdrawal from chaplaincy events reflects a protective disengagement, a common response when core beliefs about safety and institutional trust are violated, potentially hindering his ability to fulfill his perceived purpose as a student.

Moral Orienting Systems also provides insight into the experience of John, the senior administrator. His language of “delicate,” “discretion,” “cornerstone pledge” all signal an institutional privileging of campaign success, risk management, and reputational stewardship. “Life is complicated” becomes the narrative that justifies compromise. MOS cautions that coded virtues like civility and professionalism can function to maintain status quo when power is uneven. John’s actions to quiet and conceal show his complicity with the moral drift of the institution, but too often unseen is the inner conflict that may also be occurring. At risk is John’s sense of moral agency in promoting a flourishing community for all, his relationship with Isabel, the campus chaplain, with Daniel and other students, as well as other colleagues and leaders within the organization.

While John’s actions seem to be attempting to cleanse the university of immorality, putting the moral burden back on the less powerful subjects, Isabel and Daniel – how will these actions affect John in the immediate and long terms? John’s complicity, while perhaps maintaining institutional equilibrium, risks long-term erosion of his own MOS, potentially leading to a cynical detachment from previously held values or a chronic sense of inauthenticity regarding his purpose. This internal conflict between institutional demands and personal/professional ethics represents a potential erosion of his own moral coherence, impacting his long-term sense of meaning.

This manner of Moral Orienting System mapping could continue to include the donor, Mr. Harrison, other administrators, and the university itself. Complex situations like this are not exclusively contained within an acute morally injurious event but ripple out in social/relational space and time, impacting multiple subjects. Stories like this don’t end when the institution moves on, and it’s worth considering the lasting impacts this situation may generate: how does each impacted subject cope with their experience? Do they find reparative actions elsewhere that reestablish trust and meaningfulness or does this morally disorienting experience drive deepening disconnection, disillusionment, and despair? Does anyone notice, or understand the underlying cause, when Isabel leaves the university or the profession altogether, or when Daniel’s grades begin to suffer and he doesn’t return to school for the next term? Too often circumstances like this and their outcomes go misattributed as “retention issues” or “burnout” and are not accurately recognized for the complex moral disorientations that generated them.

The MOS framework moves beyond individual pathologizing to guide concrete, multi-level interventions. A truly reparative response would move well beyond individual therapy for Daniel to apply the five reparative processes for moral reorientation (**Table 3**). For Daniel, repair would involve (1) Narrative Reconstruction to re-story his experience not as a personal failure or “sensitivity” but as a systemic one, and (5) Moral Re-engagement with communities that affirm his inherent worth beyond his utility as a donor showpiece. For the staff, it necessitates (2) Moral Dialogue in a facilitated setting between Isabel and John to address the institutional betrayal. Finally, for the university administration, (3) Moral Reflection and (4) Moral Action would require a formal review of donor relations policies, addressing the systemic conditions that enabled the harm.

**Table 3:** Processes of moral reorientation: mechanisms and applications.

Reorientation process	Core mechanism (The “Why”)	Applied examples (The “How”)
1. Narrative Reconstruction	Moving from a fragmented or frozen trauma story to a coherent narrative of self-in-context. Maps shifts in beliefs and acknowledges systemic impact.	• Expressive writing exercises focusing on “before/after” moral maps. • Structured “Life Review” emphasizing values rather than just symptoms. • Oral history recording to externalize the experience.
2. Moral Dialogue & Disclosure	Shifting from individual isolation/unburdening to intersubjective co-construction of meaning. Repairs ruptured bonds through validation.	• Facilitated group therapy with peers who share the moral landscape. • “Moral Injury Circles” in institutional settings. • Structured disclosure sessions with non-judgmental moral authorities (chaplains, mentors).
3. Moral Reflection & Education	Critical examination of the systemic context to reduce self-blame. Refines moral beliefs to accommodate new realities.	• Organizational ethics education that analyzes systemic pressures. • Guided study of wisdom traditions or moral philosophy. • “Values sorting” exercises to identify conflicting institutional vs. personal norms.
4. Moral Action & Atonement	Moving from passive guilt to active agency. Restorative actions that embody reconstructed values and address conditions of harm.	• Leading or participating in policy reform advocacy. • Community service aligned with personal values. • Restorative justice circles focused on making amends to affected communities.
5. Moral Re-engagement & Belonging	Restoring a felt sense of belonging within a coherent moral community. actively finding spaces where the new moral identity is affirmed.	• Reconnecting with estranged faith or community groups. • Joining “affinity groups” based on shared lived experience. • Mentoring others entering the profession to foster a healthier moral culture.

## Conclusion

This paper has argued that current conceptualizations of moral injury and corresponding measurements are limited by their focus on individual symptoms, discrete events, and intrapsychic processes. The most psychometrically rigorous MI measures, like the MIOS and MIDS, achieve this rigor by indexing symptoms to a specific event, a design feature that increases internal validity. However, from a systems-thinking perspective, this very strength is a conceptual weakness, as it renders them less capable of capturing structural dynamics. They are excellent tools for measuring a “moral broken bone” but are ill-equipped to assess “moral osteoporosis” from a toxic moral environment, or the kinds of things that support good “moral bone health” throughout one’s life.

In response, we have proposed a reconceptualization through the lens of Zachary Moon’s Moral Orienting Systems (MOS) framework, understanding moral suffering as moral disorientation within the stabilizing interplay of meaningful beliefs, values, behaviors, and relationships that structure an individual’s worldview and decision-making. As the composite case study illustrated, the MOS framework allows for a mapping of the moral ecology itself, revealing how systemic pressures, institutional narratives, and power imbalances contribute to disorientation for multiple actors.

This paper has built a sustained case that new assessment tools are needed to resolve the field’s “precision vs. breadth” trade-off and capture the systemic, relational nature of moral suffering. In response, we are developing the Moral Orienting System Assessment (MOSA) as the logical and necessary answer to that demonstrated need. Unlike traditional instruments that produce a single severity score, the MOSA maps an individual’s position within a two-dimensional moral space defined by two orthogonal axes: (1) Moral Stress Intensity and (2) Strength of System.

The first dimension captures factors that destabilize the MOS, integrating items that measure self-condemning moral emotions (e.g., “I feel anger over being betrayed by someone whom I had trusted”), spiritual struggle (e.g., “I feel spiritual emptiness or disconnection during moral questioning”), and exposure to injurious events. The second dimension assesses the foundational sources of moral resilience and systemic coherence, targeting domains such as beliefs and values integration (e.g., “My core values are integrated into my daily decisions and interactions”), social-relational functioning (e.g., “I engage in conversations that challenge or reinforce my core moral beliefs”), and reparative action orientation (e.g., “I seek forgiveness and pursue reconciliation with those I have wronged”). By assessing these domains simultaneously, the MOSA provides a “vector” of moral orientation rather than a simple pathology score, distinguishing between individuals experiencing high stress who maintain systemic stability (resilience) and those whose systems have collapsed (disorientation), thus offering a more nuanced baseline for the clinical and organizational interventions proposed in this framework. We anticipate that the MOSA will offer a more comprehensive, strengths-based, and context-sensitive means to understand the nuances of moral suffering and identify pathways toward growth and moral reorientation.

This article is conceptual and advances a framework rather than reporting empirical findings. The case is a composite designed to illustrate application, so it should not be read as evidence of prevalence or effect size. Future studies will operationalize the construct and test discriminant validity against MIOS and MIDS, examine measurement invariance across sectors and demographic groups, and evaluate reliability, sensitivity to change, and practical utility in clinical, pastoral, and organizational settings.

Shifting towards an MOS framework and developing tools like the MOSA holds significant potential for advancing research, clinical practice, and pastoral care related to moral suffering. By embracing a more systemic, relational, and contextually attuned understanding of moral disorientation, we can foster more effective, compassionate, and potentially transformative responses. This involves not only tending to individual wounds but also addressing the moral ecologies and systemic forces that contribute to disorientation, thereby supporting the cultivation of moral resilience, wisdom, and flourishing within individuals and communities confronting the complex moral demands of our time.

## Data Availability

The original contributions presented in the study are included in the article/supplementary material. Further inquiries can be directed to the corresponding author.
